# Covalent Organic Framework-Functionalized Magnetic CuFe_2_O_4_/Ag Nanoparticles for the Reduction of 4-Nitrophenol

**DOI:** 10.3390/nano10030426

**Published:** 2020-02-28

**Authors:** Chen Hou, Dongyan Zhao, Wenqiang Chen, Hao Li, Sufeng Zhang, Chen Liang

**Affiliations:** 1 College of Bioresources Chemical and Materials Engineering, Shaanxi Provincial Key Laboratory of Papermaking Technology and Specialty Paper Development, Key Laboratory of Paper Based Functional Materials of China National Light Industry, National Demonstration Center for Experimental Light Chemistry Engineering Education, Shaanxi University of Science and Technology, Xi’an 710021, China; 1601061@sust.edu.cn (D.Z.); 1801075@sust.edu.cn (W.C.); 1701013@sust.edu.cn (H.L.); 2 Key Laboratory of Clean Pulp & Papermaking and Pollution Control of Guangxi Province, Guangxi University, Nanning 543003, China; liangchen@gxu.edu.cn

**Keywords:** magnetic covalent organic frameworks, nanocomposite, core-shell, p-nitrophenol, reduction

## Abstract

In this work, magnetic CuFe_2_O_4_/Ag nanoparticles activated by porous covalent organic frameworks (COF) was fabricated to evaluate the heterogenous reduction of 4-nitrophenol (4-NP). The core-shell CuFe_2_O_4_/Ag@COF was successfully prepared by polydopamine reduction of silver ions on CuFe_2_O_4_ nanoparticles, followed by COF layer condensation. By integrating the intrinsic characteristics of the magnetic CuFe_2_O_4_/Ag core and COF layer, the obtained nanocomposite exhibited features of high specific surface area (464.21 m^2^ g^−1^), ordered mesoporous structure, strong environment stability, as well as fast magnetic response. Accordingly, the CuFe_2_O_4_/Ag@COF catalyst showed good affinity towards 4-NP via π-π stacking interactions and possessed enhanced catalytic activity compared with CuFe_2_O_4_/Ag and CuFe_2_O_4_@COF. The pseudo-first-order rate constant of CuFe_2_O_4_/Ag@COF (0.77 min^−1^) is 3 and 5 times higher than CuFe_2_O_4_/Ag and CuFe_2_O_4_@COF, respectively. The characteristics of bi-catalytic CuFe_2_O_4_/Ag and the porous COF shell of CuFe_2_O_4_/Ag@COF made a contribution to improve the activity of 4-NP reduction. The present work demonstrated a facile strategy to fabricate COF-activated nano-catalysts with enhanced performance in the fields of nitrophenolic wastewater treatment.

## 1. Introduction

Nitrophenols are mutagenic and refractory aromatic pollutants commonly found in various industrial and agricultural wastewaters. Among the three isomeric nitrophenols, 4-nitrophenol (4-NP) is more detrimental to pose a threat to both the environment and the public [1]. Generally, two conventional clean-up techniques are used for the removal of 4-NP: (1) permanent removal of target contaminants, and (2) conversion into less- or non-toxic forms. A technique related to the latter pattern would be desirable because of the production of *p*-animophenol (4-AP), which is an important intermediate in the productions of pharmaceuticals, dyes, pigments and pesticides [2,3,4]. Therefore, high-efficiency and recycled catalysts have been desired for 4-NP reduction. 

Catalytic reduction using noble metals as catalysts have been widely employed for 4-NP reduction due to the high surface areas and the exposed active atoms. However, due to the high surface free energy, noble metals tend to agglomerate, thus leading to obvious decrease of the catalytic activities [5]. To improve the catalytic activity and stability, it would be feasible to immobilize noble metals on a matrix, especially another catalytic component, which would reduce its aggregation as well as improve the catalytic performance in contrast with monometallic counterparts [6,7].

Magnetic CuFe_2_O_4_ nanoparticles have been used in several water-treatment applications due to their desirable stability, easy separation and remarkable catalysis [8,9]. Thus, instead of single-component metals, noble metal@CuFe_2_O_4_ catalyst would reduce the aggregation of noble metal nanoparticles and maximize the synergistic effects of the bi-catalyst [10,11]. However, because of inter-particle aggregation and a non-porous structure, noble metal@CuFe_2_O_4_ nanoparticles still have disadvantages such as a limited stability and low surface area. Hence, active noble metal@CuFe_2_O_4_ nanoparticles dispersed on porous solid supports are gaining increasing interest for more effective and versatile catalysis. 

Covalent organic frameworks (COF) are an emerging type of porous materials being extensively studied due to their excellent properties and broad applications [12,13,14,15]. In comparison with their materials similar to metal organic frameworks (MOF), robust covalent bonds on COF enable it to overcome the problems of water and moisture instability [16,17]. The fascinating features such as low density, high and regular porosity, tunable pore size [18,19], render them promising candidates in diverse applications in catalysis, gas storage, adsorption, optoelectricity and chemical sensors [20,21,22,23,24]. Recently, some research has reported that COF can heterogeneously nucleate and grow on the surface of different matrices to construct core-shell structure composite materials (graphene, carbon nanotubes, Fe_3_O_4_ and alumina, etc.) [25,26,27,28]. Incorporation of the merits of COF and nanosized components for core-shell structure nanocomposites synthesis, the aggregation of nanosized cores can be effectively impeded while allowing facile surface modification. Therefore, based on the feasibly tuned properties by the in-built covalent bond architecture, it can be anticipated that COF can be used as more suitable scaffolds than other kinds of porous materials for fabricating core-shell structured noble metal@CuFe_2_O_4_ nanocomposite.

Herein, a facile synthesis strategy of core-shell structure nanocomposite was developed to integrate magnetic CuFe_2_O_4_/Ag nanoparticles and porous COF for 4-NP reduction. Due to the outstanding stability and chemical robustness [29], TAPB-DMTP (1,3,5-tris(4-aminophenyl)benzene and 2,5-dimethoxyterephaldehyde, respectively) was utilized as the COF shell material coated on the surface of CuFe_2_O_4_/Ag nanoparticles via the polyvinylpyrrolidone (PVP)-assisted encapsulation strategy [30]. Meanwhile, the unique π-π electron structure of COF and strong magnetic property of magnetic CuFe_2_O_4_/Ag@COF composites can provide both affinity sites and the adsorption forces for reaction targets, as well as facilitate the rapid and easy separation. In addition, the high surface area of the TAPB-DMTP coating ensures high loading capacity of the reaction substrates. Accordingly, the as-prepared porous CuFe_2_O_4_/Ag@COF nanocomposite exhibited a high surface area, large number of affinity sites and strong magnetic property owing to the combination of the merits of CuFe_2_O_4_/Ag and TAPB-DMTP. By virtue of the unique features, CuFe_2_O_4_/Ag@COF acted as the recyclable catalyst for 4-NP reduction by NaBH_4_ and showed enhanced catalytic activities and stability. 

## 2. Experimental

### 2.1. Materials

1,3,5-tris(4-aminophenyl)benzene (TAPB), 2,5-dimethoxyterephaldehyde (DMPT) and dopamine hydrochloride (DA·HCl), polyethylene glycol (PEG-6000) and polyvinylpyrrolidone (PVP, Mw = 5500) were purchased from Aladdin Co. Ltd. (China). Cupric chloride anhydrous (CuCl_2_), ferric chloride hexahydrate (FeCl_3_·6H_2_O), ammonium acetate (NH_4_Ac), ethylene glycol (EG), silver nitrate (AgNO_3_), NaCH_2_COOH (NaAc), CH_3_COOH (HAc), 4-nitrophenol (4-NP), sodium borohydride (NaBH_4_), and other chemicals and reagents were purchased from Sinopharm Chemical Reagent Co. Ltd. (Xi’an, China). 

### 2.2. Measurements

Sample morphologies were characterized by field-emission scanning electron microscopy (SEM, Hitachi S-4800, Hitachi, Japan) and transmission electron microscopy (TEM, TECNAI G^2^ TF20, Hillsboro, OH, USA). Fourier transform infrared (FTIR, Bruker VERTEX 70, Karlsruhe, Germany) spectra were obtained with a wavelength range of 4000 to 400 cm^−1^. Powder X-ray diffraction spectra (XRD, Bruker, D8-Advance, Karlsruhe, Germany) were measured with Cu Kα radiation (λ = 1.542 Å) in the angle range 5–70° (2*θ*). The specific surface area and pore size distribution were calculated by the Brunauer–Emmett–Teller (BET, ASAP2460, Atlanta, GA, USA) and Barrett–Joyner–Halanda (BJH) methods. Thermogravimetric analysis (TGA) was recorded on a TG apparatus (NETZSCH STA 449F3-1053-M, Selb, Germany) by heating the sample in the range 30–800 °C at a constant rate of 10 °C  min^−1^ under a N_2_ atmosphere. Magnetic hysteresis loops at room temperature were obtained using a vibrating sample magnetometer VSM 7304 (Lakeshore, Columbus, OH, USA). The chemical composition of CuFe_2_O_4_/Ag and CuFe_2_O_4_/Ag@COF were characterized by X-ray photoelectron spectroscopy (XPS, AXIS SUPRA, Manchester, UK). The absorbance of the reaction solution was determined by ultraviolet-visible (UV-Vis) spectra (Shimadzu UV-2501 PC spectrometer, Suzhou, China).

### 2.3. Synthesis of CuFe_2_O_4_/Ag Nanoparticles 

CuFe_2_O_4_ nanoparticles were synthesized according to the reported method [31]. Then the Ag nanoparticle was assembled on CuFe_2_O_4_ according to a polydopamine (PDA)-assisted reduction process [32]. Typically, 30 mg of CuFe_2_O_4_ nanoparticles were dispersed in 25 mL Tris buffer solution (10 mM, pH = 8.5), followed by adding 50 mg of DA·HCl into the mixture and mechanically stirred for 2 h at room temperature. Then the black powder was collected by a magnet, washed with ethanol and deionized water. For the preparation of CuFe_2_O_4_/Ag nanoparticles, silver ammonia solution was firstly prepared by adding ammonia aqueous solution (2 wt%) into 10 mg·mL^−1^ AgNO_3_ solution until the brown precipitation was just dissolved. Then 50 mg of PDA-modified CuFe_2_O_4_ were added to 25 mL silver ammonia solution, and the mixture was mechanically stirred for 6 h at room temperature. The product was collected with a magnet and washed with ethanol and deionized water, then dried under vacuum at 60 °C over night.

### 2.4. Preparation of Core-Shell Structured CuFe_2_O_4_/Ag@COF Nanocomposites

The synthesis of core-shell structured CuFe_2_O_4_/Ag@COF nanocomposites was carried out as a modified method [33]. Firstly, CuFe_2_O_4_/Ag nanoparticles were stabilized with PVP. In brief, the CuFe_2_O_4_/Ag nanoparticles (50 mg) were suspended in water (50 mL), and a solution of PVP (25 mg) in water (50 mL) was added dropwise to the above solution under stirring. The mixture was further stirred at room temperature for 12 h, washed three times with methanol, and final dispersed in methanol (10 mL). Secondly, the PVP-stabilized CuFe_2_O_4_/Ag nanoparticles were sonicated for 30 min under stirring, and 4 mL 1,4-dioxane-butanol (v/v = 1/1) containing TAPB (0.03 mmol) and DMTP (0.045 mmol) was added to the suspension. After that, aqueous acetic acid (12 M, 0.15 mL) was slowly appended to the mixture. Then, the reaction was allowed to conduct at room temperature for 2 h. After another aqueous acetic acid (12 M, 0.45 mL) was added to the suspension, the reaction was further stirred at 70 °C for 24 h. The product (CuFe_2_O_4_/Ag@COF) was naturally cooled to room temperature and collected with the help of a magnet. After being washed with tetrahydrofuran and acetone several times, the product was dried in vacuum at 80 °C overnight. 

The CuFe_2_O_4_@COF nanocomposite was prepared according to the above method by adding CuFe_2_O_4_ nanoparticles.

### 2.5. Catalytic Reduction of 4-Nitrophenol (4-NP)

Catalytic activities of the CuFe_2_O_4_/Ag@COF nanocomposite were evaluated by UV-Vis spectroscopy through the reduction of 4-NP to 4-AP at room temperature, using NaBH_4_ as a reductant. 200 μL of 4-NP solution (5 mM), 0.45 mL of freshly prepared NaBH_4_ aqueous solution (200 mM), and 2.35 mL deionized water were added into a standard quartz cuvette. The mixture solution turns from light yellow to bright yellow. Subsequently, 2 mg of CuFe_2_O_4_/Ag@COF was dispersed to the above mixture. Then the intensity of the absorption peak at 400 nm was monitored by UV-Vis spectroscopy as a function of time. The as-prepared CuFe_2_O_4_ nanoparticles, CuFe_2_O_4_/Ag nanoparticles, and CuFe_2_O_4_@COF nanocomposite were also applied for 4-NP reduction under the same conditions. For the catalyst recycling test, the catalyst was collected with an extra magnet after each cycle, and another 4-NP and NaBH_4_ solution was added for the next catalytic cycle. The UV-Vis absorption of reaction solution was measured at 400 nm.

## 3. Results and Discussion 

### 3.1. Fabrication and Characterization of CuFe_2_O_4_/Ag@COF Nanocomposite

**Scheme 1:**
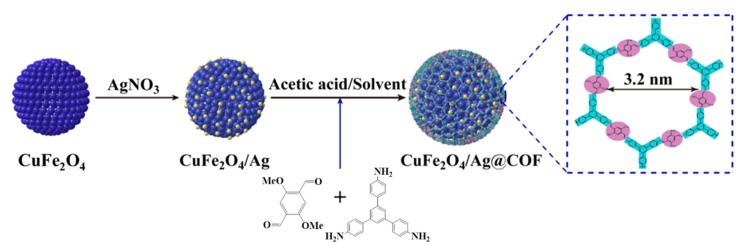
Schematic representation of the synthesis of core-shell magnetic CuFe_2_O_4_/Ag@COF nanocomposite.

The magnetic CuFe_2_O_4_/Ag@COF was synthesized by using CuFe_2_O_4_/Ag as a core, and TAPB-DMTP COF as a shell (Scheme 1). CuFe_2_O_4_/Ag was synthesized by depositing Ag on CuFe_2_O_4_ nanoparticles, which was assisted by PDA reduction. This bi-catalyst composed of CuFe_2_O_4_ and in situ synthesized Ag nanoparticles would accelerate the 4-NP reduction [34]. The COF shell encapsulation process was assisted by polyvinylpyrrolidone (PVP)-stabilized CuFe_2_O_4_/Ag strategy. This is because it has been proved that the amphiphilic PVP can stabilize the nanoparticles in the reaction solution and enhance the affinity between apolar groups of PVP and organic linkers [30]. At last, the TAPB-DMTP was assembled on the surface of CuFe_2_O_4_/Ag based on Schiff-base reaction to obtain the core-shell CuFe_2_O_4_/Ag@COF nanocompsite [33].

**Figure 1:**
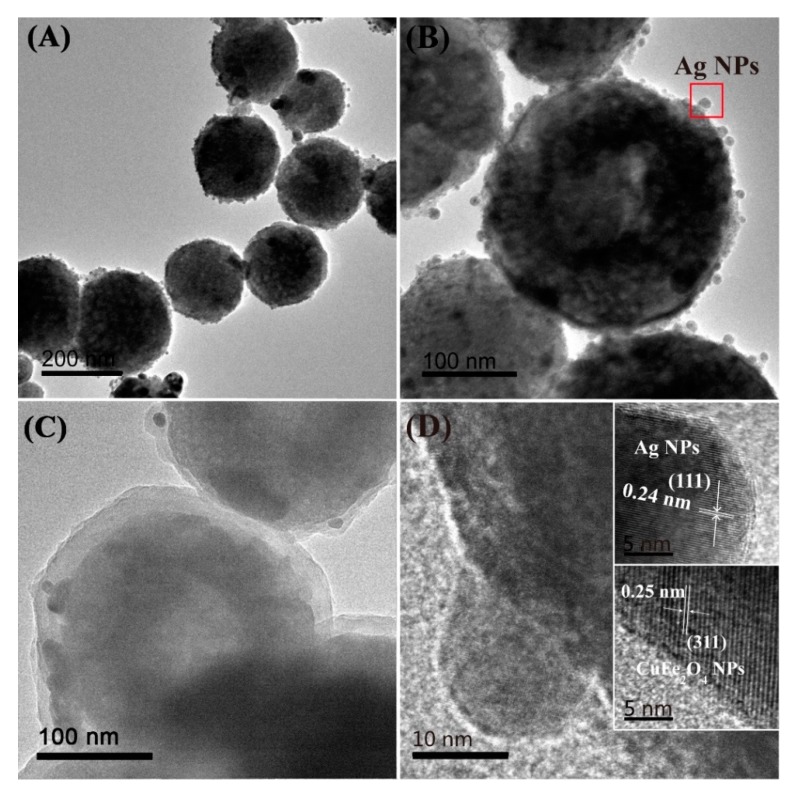
Transmission electron microscope (TEM) images of the CuFe_2_O_4_/Ag (**A**) and (**B**); CuFe_2_O_4_/Ag@COF (**C**); and HRTEM image of CuFe_2_O_4_ and Ag (**D**).

The core-shell structure and morphologies of the as-prepared CuFe_2_O_4_/Ag@COF nanocomposite was verified by transmission electron microscopy (TEM) and scanning electron microscopy (SEM). Figure 1A,B revealed that the as-prepared CuFe_2_O_4_/Ag nanoparticles exhibited good monodispersity, and the CuFe_2_O_4_ were spherical in shape with an average diameter of 200 nm. After being deposited with Ag nanoparticles, small Ag nanoparticles (∼20 nm) were densely and uniformly assembled on the surface of CuFe_2_O_4_. After being coated with a COF layer, the COF shell (brighter) of TAPB-DMTP networks with a rough surface was formed and the thickness of ca. 30 nm surrounding the CuFe_2_O_4_/Ag core (darker) was clearly observed (Figure 1C). Meanwhile, at the edges of CuFe_2_O_4_/Ag@COF spheres consisted of domains that contain regular channels along the radial direction (Figure S1). It is due to the self-assembly of the rod-like crystallites of the COF shell [35] and revealed a highly ordered porous structure of the CuFe_2_O_4_/Ag@COF. Figure 1D displayed the high-resolution TEM images (HRTEM) and the corresponding fast Fourier transform (FFT) pattern of the CuFe_2_O_4_/Ag@COF nanocomposite. The spacing of the crystallographic plane of the Ag nanoparticles is 0.24 nm, which could be assigned to the (111) crystal planes of face-centered cubic (fcc) bulk Ag (0.235 nm). A clear inter-planar spacing of the lattice fringes is 0.25 nm, corresponding to the (311) crystal planes of CuFe_2_O_4_ with cubic spinel structure. 

**Figure 2:**
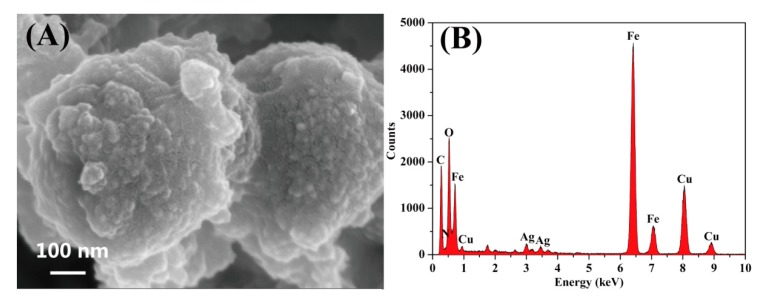
(**A**) Scanning electron microscope (SEM) image of CuFe_2_O_4_/Ag@COF; (**B**) energy-dispersive X-ray spectroscopy (EDX) pattern of CuFe_2_O_4_/Ag@COF.

Furthermore, the SEM image of CuFe_2_O_4_/Ag@COF also shows that the nanocomposite consisted of spheres with a rough and coarse surface (Figure 2A). The obtained nanocomposite is somewhat visibly sticky and exhibited convex domains due to the presence of porous TAPB-DMTP. To confirm the elemental composition of the CuFe_2_O_4_/Ag@COF, the EDX elemental mapping confirmed the presence of Cu, Fe, Ag, C, N, and O element (Figure 2B).

**Figure 3:**
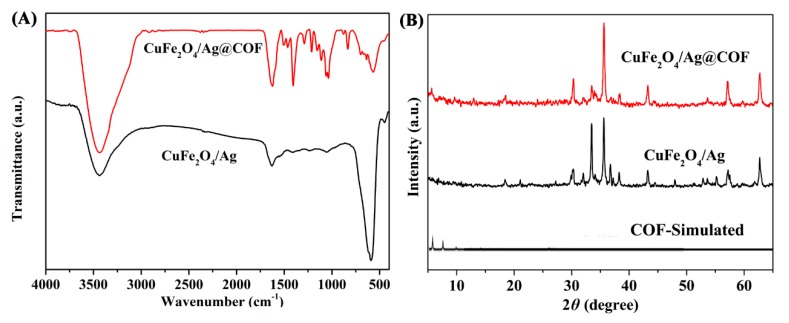
(**A**) Fourier transform infrared (FTIR) spectra of CuFe_2_O_4_/Ag and CuFe_2_O_4_/Ag@COF; (**B**) X-ray diffraction (XRD) patterns of CuFe_2_O_4_/Ag and CuFe_2_O_4_/Ag@COF.

Fourier transform infrared (FTIR) spectrosopy was performed to confirm the successful preparation of the CuFe_2_O_4_/Ag@COF nanocomposite (Figure 3A). In the spectrum of CuFe_2_O_4_/Ag, the absorption broad band at 3430 cm^−1^ represented the stretching mode of –OH groups and another peak at 1620 cm^−1^ corresponded to the bending vibration of H_2_O. In the lower zone, peak at 590 cm^−1^ can be related to the vibration of the metal (tetrahedron)-oxygen (M–O), in which the atom M can be copper or iron. Another absorption peak at 428 cm^−1^ was referred to the vibration of octahedral sites of spinel-type oxide [36]. Compared with CuFe_2_O_4_/Ag, similar adsorption bands were observed in the spectrum of the as-prepared CuFe_2_O_4_/Ag@COF nanocomposite. New peaks at 1460 cm^−1^, 1501 cm^−1^ and 1405 cm^−1^ were assigned to the aromatic C–C ring stretch and methoxy group stretching the of TAPB-DMTP, respectively. Furthermore, the characteristic peaks at 3430 cm^−1^ and 1620 cm^−1^ became strong and sharp due to the existence of N–H bonds and C=N vibration of COF coating, validating the successful formation of COF shell via Shiff-base condensation reaction.

The crystallinity of CuFe_2_O_4_/Ag and CuFe_2_O_4_/Ag@COF was investigated by XRD measurements and the result was shown in Figure 3B. As shown in Figure 3B, the characteristic diffraction peaks at 2θ = 30.2°, 35.5°, 43.4°, 57.3° and 62.6° corresponding to the (220), (311), (400), (511) and (440) crystal planes of CuFe_2_O_4_ [36]. The pattern at 38.0°, 44.2° and 64.4° can be attributed to the (111), (200) and (220) crystalline planes of the synthesized Ag [37]. After the coating of TAPB-DMTP COF, the moderate new peaks at 2θ = 5.60° and 7.4°, and weak peak at 9.7° belonging to the TAPB-DMTP was observed, which is consistent with that of TAPB-DMTP COF previously reported [29]. These results suggested that the CuFe_2_O_4_/Ag@COF nanocomposite exhibited the crystalline structure feature of magnetic CuFe_2_O_4_/Ag and TAPB-DMTP COF. 

**Figure 4:**
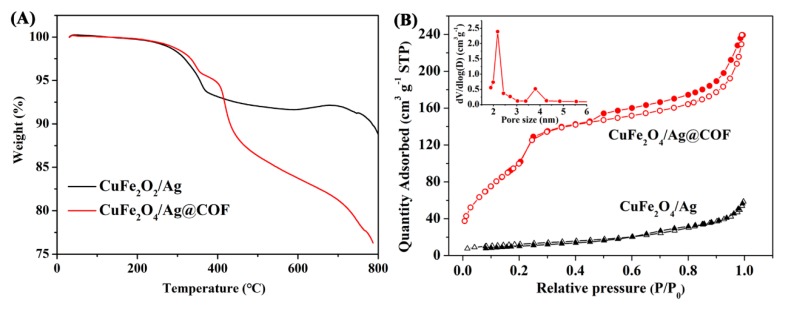
(**A**) Thermogravimetric analysis (TGA) curves of CuFe_2_O_4_/Ag and CuFe_2_O_4_/Ag@COF (**B**) N_2_ adsorption-desorption isotherms of CuFe_2_O_4_/Ag and CuFe_2_O_4_/Ag@COF (the inset shows pore size distribution of CuFe_2_O_4_/Ag@COF).

TGA was further recorded to confirm the content of TAPB-DMTP coatings on the magnetic CuFe_2_O_4_/Ag (Figure 4A). As for CuFe_2_O_4_/Ag nanoparticles, a distinct weight-loss step of 1.7 wt% up to ca. 300 °C was observed, which can be attributed to the volatilization of the absorbed water and solvent molecule. Another sharply weight loss profile (5.0 wt%) in the range of 300–400 °C was observed due to the decomposition of PDA. Then it remained constant until the temperature rose to 800 °C, and demonstrated a remarkable thermo-stability of CuFe_2_O_4_/Ag. Compared with CuFe_2_O_4_/Ag, the CuFe_2_O_4_/Ag@COF nanocomposite had almost 14 wt% weight loss in the range of 400–700 °C, implying the decomposition of TAPB-DMTP and further demonstrated the presence of TAPB-DMTP COF shell. Meanwhile, the CuFe_2_O_4_/Ag@COF also exhibited excellent thermal stability in nitrogen up to 300 °C.

The pore structure of the CuFe_2_O_4_/Ag@COF was further evaluated by N_2_ adsorption-desorption at 77 K (Figure 4B). The isotherm of CuFe_2_O_4_/Ag presented a typical II characteristics, indicating the obviously non-porous structure of the solid sphere. Compared to CuFe_2_O_4_/Ag, a typical type IV isotherm was observed by CuFe_2_O_4_/Ag@COF, which is indicative of a mesoporous character. From pore size distribution curve (the inset of Figure 4B), the CuFe_2_O_4_/Ag@COF contained an average pore size of 3.15 nm with narrow sized distribution, which was well in agreement with theoretical value of the bulk COF (3.2 nm) [33]. In addition, the prepared CuFe_2_O_4_/Ag@COF gave an enhanced BET surface area and pore volume of 464.21 m^2^ g^−1^ and 0.396 m^3^ g^−1^ (38.60 m^2^ g^−1^ and 0.0862 m^3^ g^−1^ for CuFe_2_O_4_/Ag), respectively (Table S1). The high external surface area with mesoporous structure indicates the superior concentration performance for the substrates, and thus made it possible for CuFe_2_O_4_/Ag@COF to facilitate efficient catalysis reduction of 4-NP. 

The vibrating sample magnetization (VSM) curves of CuFe_2_O_4_/Ag nanoparticle and CuFe_2_O_4_/Ag@COF nanocomposite are shown in Figure S2. All the materials obtained exhibited a superparamagnetic nature due to no obvious coercivity and remanence. After being assembled with TAPB-DMTP, the saturation magnetization of CuFe_2_O_4_/Ag@COF decreased from 62.7 emu g^−1^ to 35.1 emu g^−1^. However, very fast aggregation (about 1 min) of CuFe_2_O_4_/Ag@COF from the homogeneous dispersion was observed with the help of an extra magnet (as shown in the bottom-right inset of Figure S2).

**Figure 5:**
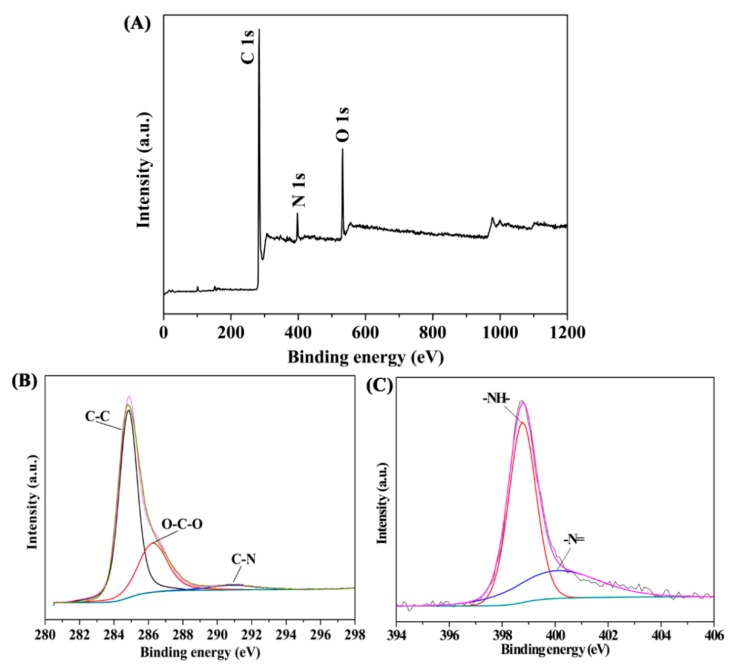
X-ray photoelectron spectroscopy (XPS) spectra of CuFe_2_O_4_/Ag@COF: (**A**) full survey spectrum, (**B**) C 1s, and (**C**) N 1s regions.

To further analyze the chemical composition of the magnetic CuFe_2_O_4_/Ag@COF, XPS was carried out. In Figure 5A, the wide scan spectra of the magnetic CuFe_2_O_4_/Ag@COF displayed photoelectron lines at binding energies of 285, 402, and 528 eV, which can be assigned to C 1s, N 1s, and O 1s, respectively. The disappearing Fe, Cu, Ag peaks in the wide scan spectra indicating the inner CuFe_2_O_4_/Ag core was coated with a shell which was thicker than the analysis depth of XPS (~10 nm). The C 1s core-level photoelectron spectrum was split into three peaks located at 284.6, 286.3, and 290.8 eV, respectively (Figure 5B), which can be attributed to C–C/C=C, C–N, and C=O, respectively. The binding energies observed at Figure 5C showed the appearance of –NH– and –N= with their characteristic peaks at 398.4 and 400.0 eV. These results confirmed the successful formation of the COF shell.

### 3.2. Catalytic Reduction of 4-NP

The as-prepared CuFe_2_O_4_/Ag@COF nanocomposite has advantages of large surface area, rapid magnetic response, abundant catalytic sites, which can make sure the high catalytic performance and the catalyst stability towards 4-NP reduction. The catalytic performance of CuFe_2_O_4_/Ag@COF was tested for reducing 4-NP in the presence of excess NaBH_4_ in water. The reaction kinetics were monitored by UV-vis absorption spectroscopy of the reaction mixture after adding the catalyst. Notably, the absorption peak of 4-nitrophenol shifts from 319 to 400 nm (the aqueous solution turns from light yellow to bright yellow rapidly) after adding NaBH_4_ due to the formation of 4-nitrophenolate ions in the alkaline medium. 

**Figure 6:**
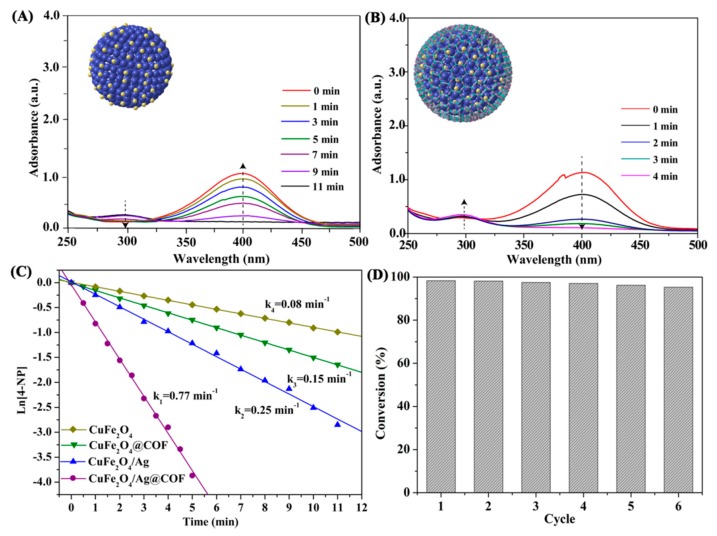
(**A**) Typical time-dependent evolution of ultraviolet-visible (UV-Vis) spectra for the catalytic reduction of 4-nitrophenol (4-NP) to *p*-animophenol (4-AP) by CuFe_2_O_4_/Ag; (**B**) typical time-dependent evolution of UV-Vis spectra for the catalytic reduction of 4-NP to 4-AP by CuFe_2_O_4_/Ag@COF; (**C**) relationship of the ln[4-NP] and the reaction time t for the reduction of 4-NP to 4-AP over CuFe_2_O_4_, CuFe_2_O_4_@COF, CuFe_2_O_4_/Ag and CuFe_2_O_4_/Ag@COF; (**D**) the reusability of CuFe_2_O_4_/Ag@COF for the reduction of 4-NP by NaBH_4_.

Figure 6A,B showed the successive UV-vis spectra of 4-nitrophenolate catalyzed by CuFe_2_O_4_/Ag and CuFe_2_O_4_/Ag@COF in the presence of NaBH_4_, respectively. The absorption peak at 400 nm was observed to decrease in intensity and a new peak at ~300 nm increased along with the reaction time, suggesting the reduction of 4-NP to give 4-AP as the sole product [38]. Figure 6A shown that the catalytic reduction with CuFe_2_O_4_/Ag nanoparticles could be completed within 11 min. When CuFe_2_O_4_/Ag@COF was used as the catalyst (Figure 6B), a significant catalytic activity was observed and conversion of 4-NP to 4-AP was finished within 4 min. We can speculate that the momentous activity shown by CuFe_2_O_4_/Ag@COF over CuFe_2_O_4_/Ag own to the porous COF matrix, which provided a large surface area of the encapsulated CuFe_2_O_4_/Ag nanoparticles and high particle number per unit mass for the catalyst. The increased fraction of the substrates at the surface of CuFe_2_O_4_/Ag@COF led to a significantly higher catalytic activity.

Since the concentration of BH_4_^−^ added in the system is in excess compared to the concentration of 4-NP, pseudo-first-order kinetics is expected for the catalytic reaction under this circumstance (Figure 6C). The pseudo-first-order kinetics can be described as:

ln(*C*_t_/*C*_0_) = −*k*t (1)

where *C*_t_ is the concentration of 4-NP at time t, *C*_0_ is the initial concentration of 4-NP, and *k* is the rate constant. From Figure 6C we can see all composite samples can catalyze 4-NP reduction but display different activities. Basically, single-catalyst showed lower catalytic activity than the dual-catalyst. For example, CuFe_2_O_4_@COF had a decreased catalytic activity (with the rate constant of 0.15 min^−1^) and much lower activity for CuFe_2_O_4_ (with the rate constant of 0.08 min^−1^), respectively, in comparison with that of CuFe_2_O_4_/Ag (0.25 min^−1^). Moreover, when dual-catalyst was applied, the catalytic performances of the resulting catalysts were inherently influenced by the crystal COF shell. For example, CuFe_2_O_4_/Ag@COF displayed a higher catalytic activity (with the rate constant of 0.77 min^−1^) than that of CuFe_2_O_4_/Ag. Specifically, CuFe_2_O_4_/Ag@COF exhibited the highest activity, which was 3, 5 and 10 times higher than that of CuFe_2_O_4_/Ag, CuFe_2_O_4_@COF and CuFe_2_O_4_, respectively. It can be assumed that the sluggish reaction kinetics of the above three kinds of catalysts (CuFe_2_O_4_/Ag, CuFe_2_O_4_@COF and CuFe_2_O_4_) highlighted the utility of the dual-catalyst and ordered mesoporous COF shell. This result also showed superior catalytic activity compared to the composite of noble metals (such as Ag, Pd, and Au nanoparticles) embedded in other porous matrix tested under the similar conditions (Table S2). 

Additionally, the reusability of CuFe_2_O_4_/Ag@COF was further displayed. With high saturation magnetization, the CuFe_2_O_4_/Ag@COF can be easily separated in a few seconds using an external magnetic field. Figure 6D displayed the conversion for the reduction of 4-NP in an estimated time span of 4 min. The result displayed excellent reusability of the catalyst for more than 6 catalytic cycles with yields over 95%, indicating excellent reusability and stability towards the as-prepared CuFe_2_O_4_/Ag@COF nanocomposite. The slightly decreased conversions in the later catalysis cycles are presumably caused by the loss of catalyst during the washing process between cycles. The SEM image manifested that the structure of the recycled CuFe_2_O_4_/Ag@COF was preserved and no obvious aggregation was observed, further confirming the excellent stability of the prepared catalyst (Figure S3). Hence, the prominent catalytic activity and high stability of CuFe_2_O_4_/Ag@COF can be attributed to the highly stable mesoporous architecture (TAPB-DMTP), which held the encapsulated nanoparticles to a high extent and provided docking sites for 4-NP.

### 3.3. Plausible Mechanisms

**Scheme 2:**
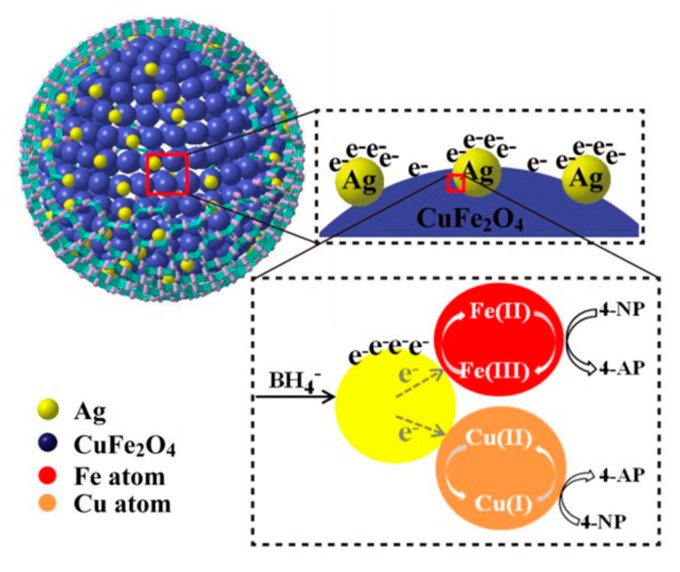
The mechanism of the reduction of 4-NP to 4-AP by NaBH_4_ in the presence of CuFe_2_O_4_/Ag@COF.

Scheme 2 depicts the performance of CuFe_2_O_4_/Ag@COF as catalyst in reducing 4-NP to 4-AP by NaBH_4_. In aqueous medium, NaBH_4_ was first diffused through the mesoporous COF shell and adhered onto the surface of CuFe_2_O_4_/Ag, then electrons transferred from CuFe_2_O_4_/Ag to the adsorbed BH_4_^−^ with hydride ions’ production. Simultaneously, the 4-NP were adsorbed onto the mesoporous COF shell via π-π stacking interactions because 4-NP is π-rich in nature [38]. Subsequently, the hydrogen atom formed from the hydride ions attacked 4-NP molecules to reduce them based on electron transfer to the CuFe_2_O_4_/Ag nanoparticles, forming the product 4-AP [38]. The surface of dual-catalyst CuFe_2_O_4_/Ag is presumed to play a role in electrically connecting two adsorbates through the surface so that electrons can be transferred from the oxidation site (NaBH_4_) to the reduction site (4-NP). Such preconcentration of BH_4_^−^ and 4-NP in the COF shell would result in more frequent contact of the inner CuFe_2_O_4_/Ag catalyst with reactants, thereby facilitating a highly efficient catalysis. Moreover, it has been revealed that CuFe_2_O_4_ with ‘d^n^’ (n = 5–9) electronic configuration exhibited better catalytic activity for reduction of nitrophenol [30]. Specifically, both Cu^2+^ and Fe^3+^ ions present in the octahedral sites exposed on the surface of particles, thus the reduction of hydride ions would be accelerated by the enhanced electron transfer boosted by Ag and neighboring CuFe_2_O_4_ nanoparticles (Scheme 2).

## 4. Conclusions

In summary, we have presented a facile and efficient strategy to construct core-shell magnetic CuFe_2_O_4_/Ag@COF nanocomposite. The obtained nanocomposite not only retained its high crystallinity, large surface area and thermal stability of the COF shell, but held high catalytic properties and strong magnetic effect of magnetic CuFe_2_O_4_/Ag nanoparticles. By virtue of these merits, the CuFe_2_O_4_/Ag@COF exhibited enhanced catalytic activity, robust recyclability and stability for the catalytic reduction of 4-NP by NaBH_4_. Specifically, the apparent reaction rate of CuFe_2_O_4_/Ag@COF was 3 times higher than that by CuFe_2_O_4_/Ag, which can be ascribed to the enhanced CuFe_2_O_4_/Ag dispersion and the strengthened interfacial interaction. Moreover, the magnetic CuFe_2_O_4_/Ag@COF can be recycled easily by a magnet and exhibited robust reusability for six cycles with minimal loss of catalytic activity but held its structural integrity. Encouraged by the effective catalysis of 4-NP with this nanocomposite, it can be expected that the rational design of the promising catalytic material with magnetic platforms and a tunable porous COF shell will be developed and applied for many redox-active environmental contaminants.

## Supplementary Materials

The following are available online at https://www.mdpi.com/2079-4991/10/3/426/s1: Figure S1: TEM image of the CuFe_2_O_4_/Ag@COF, Figure S2: Magnetic hysteresis loops of CuFe_2_O_4_/Ag and CuFe_2_O_4_/Ag@COF (the inset shows the magnetic separation behavior of CuFe_2_O_4_/Ag@COF in aqueous solution), Figure S3: SEM image of the recycled CuFe_2_O_4_/Ag@COF after six times reuse, Table S1: Nitrogen adsorption-desorption data of CuFe_2_O_4_/Ag and CuFe_2_O_4_/Ag@COF, Table S2: Comparison of *k* value of different catalytic systems for the reduction of 4-NP (298K).
