# Supplementary material for: Covalent Organic Framework-Functionalized Magnetic CuFe2O4/Ag Nanoparticles for the Reduction of 4-Nitrophenol
Source: Nanomaterials (Basel). 2020 Feb 28;10(3):426. doi: 10.3390/nano10030426 (PMC7152833; doi:10.3390/nano10030426)
Supplement: Supplementary file 1 [file nanomaterials-10-00426-s001.pdf]

Supporting Information.

Covalent Organic Framework-Functionalized Magnetic $CuFe_2O_4$/Ag Nanoparticles for the Reduction of 4-Nitrophenol

Chen Hou [1,*], Dongyan Zhao [1], Wenqiang Chen [1], Hao Li [1], Sufeng Zhang [1,*] and Chen Liang [2]

[1] College of Bioresources Chemical and Materials Engineering, Shaanxi Provincial Key Laboratory of Papermaking Technology and Specialty Paper Development, Key Laboratory of Paper Based Functional Materials of China National Light Industry, National Demonstration Center for Experimental Light Chemistry Engineering Education, Shaanxi University of Science and Technology, Xi'an 710021, China; 1601061@sust.edu.cn (D.Z.); 1801075@sust.edu.cn (W.C.); 1701013@sust.edu.cn (H.L.)

[2] Key Laboratory of Clean Pulp & Papermaking and Pollution Control of Guangxi Province, Guangxi University, Nanning 543003, China; liangchen@gxu.edu.cn

* Correspondence: houchen@sust.edu.cn (C.H.); sufengzhang@126.com (S.Z.); Tel.: +86-1829-207-8770 (C.H.)

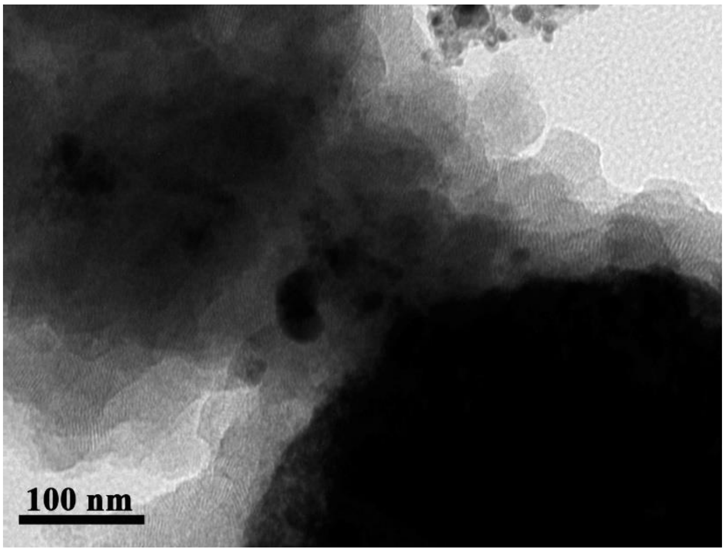

Figure S1 TEM image of the $CuFe_2O_4$/Ag@COF.

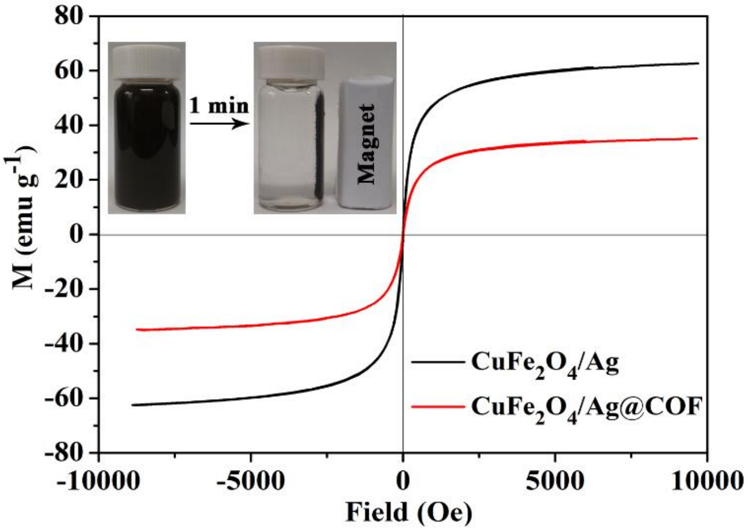

Figure S2. Magnetic hysteresis loops of $CuFe_2O_4$/Ag and $CuFe_2O_4$/Ag@COF (the inset shows the magnetic separation behavior of $CuFe_2O_4$/Ag@COF in aqueous solution).

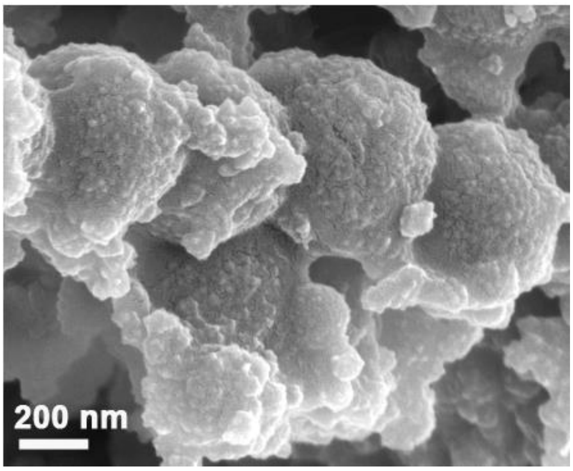

Figure S3. SEM image of the recycled $CuFe_2O_4$/Ag@COF after six times reuse.

Table S1. Nitrogen adsorption-desorption data of $CuFe_2O_4$/Ag and $CuFe_2O_4$/Ag@COF.

	BET surface area (m^2 g^{-1})	Pore volume (cm^3 g^{-1})	Pore size (nm)
$CuFe_2O_4$/Ag	38.60	0.0862	8.98
$CuFe_2O_4$/Ag@COF	464.21	0.396	3.15

Table S2. Comparison of *k* value of different catalytic systems for the reduction of 4-NP (298K).

Entry	Nanocatalysts	*k* (min^{-1})	Reference
1	Ag/C	0.33	[1]
2	Fe_3O_4@SiO_2@Ag	0.52	[2]
3	Fe_3O_4@PDA-Pd@[$Cu_3(btc)_2$]	0.72	[3]
4	Au/TAPB-DMTP-COF	0.46	[4]
5	Au@TpPa-1	0.25	[5]
6	$CuFe_2O_4$/Ag@COF	0.77	This work

References

[1] Yue, C.; Tu, J.; Wang, M. One-pot synthesis of ordered mesoporous silver nanoparticle/carbon composites for catalytic reduction of 4-nitrophenol. *J. Colloid Interf. Sci* **2014**, *423*, 54-59, DOI 10.1016/j.jcis.2014.02.029.

[2] Zhang, K.; Wang, C.; Rong, Z.; Xiao, R.; Zhou, Z.; Wang, S. Silver coated magnetic microflowers as an efficient and recyclable catalyst for catalytic reduction. *New J. Chem* **2017**, *41*, 14199-14208, DOI 10.1039/c7nj02802d.

[3] Ma, R.; Yang, P.; Ma, Y. Facile Synthesis of Magnetic Hierarchical Core-Shell Structured Fe_3O_4@PDA-Pd@MOF Nanocomposites: Highly Integrated Multifunctional Catalysts. *ChemCatChem* **2018**, *10*, 1446-1454, DOI 10.1002/cctc.201701693.

[4] Shi, X.F.; Yao, Y.J.; Xu, Y.L.; Liu, K.; Zhu, G.S.; Chi, L.F.; Lu, G. Imparting Catalytic Activity to a Covalent Organic Framework Material by Nanoparticle Encapsulation. *ACS Appl. Mater. Interfaces* **2017**, *9*, 7481-7488, DOI 10.1021/acsami.6b16267.

[5] Pachfule, P.; Kandambeth, S.; Díaz, D.D. Highly stable covalent organic framework-Au nanoparticles hybrids for enhanced activity for nitrophenol reduction. *Chem. Commun* **2014**, *50*, 3169-3172, DOI 10.1039/c3cc49176e.
